# Experimental Investigation on Hygroscopic Aging of Glass Fiber Reinforced Vinylester Resin Composites

**DOI:** 10.3390/polym14183828

**Published:** 2022-09-13

**Authors:** Weiping He, Xu Li, Piao Li, Shirui Fang, Anxin Ding

**Affiliations:** 1 Wuhan Second Ship Design and Research Institute, Wuhan 430061, China; 2 School of Materials Science and Engineering, Wuhan University of Technology, Wuhan 430070, China; 3 State Key Laboratory of Advanced Technology for Materials Synthesis and Processing, Wuhan University of Technology, Wuhan 430070, China

**Keywords:** glass fiber reinforced polymer (GFRP), vinylester resin, hygroscopic aging, moisture absorption

## Abstract

The hygroscopic behavior of vinylester resin and high strength glass fiber reinforced vinylester resin composites were examined here, including weight change and the resulting degradation of mechanical properties. The prepared resin and composites specimens were immersed in deionized water and artificial seawater with an applied temperature of 70 °C, and then the specimens were weighed at specified time intervals in combination with the observation of surface morphologies using a scanning electron microscope. Identification of variations of functional groups was also carried out using Fourier-transform infrared spectroscopy. Meanwhile, the mechanical properties for resin and the composite specimens were tested periodically. The observations on surficial morphologies and the test on weight change display that the vinylester resin hydrolyzes seriously after immersion in deionized water, and that the embedment of glass fiber effectively inhibits the moisture absorption and hydrolysis for resin matrix in composites. The results from the mechanical properties test reveal that the tensile strength of pure resin decreases by 35.3% after 7 days’ immersion and keeps small fluctuation in the sequent immersion duration. However, the compressive strength of pure resin consistently dwells at 100 ± 2 MPa during immersion. After immersion for 90 days, the tensile strength decreases by 28.5% and 38.4%, the compressive strength reduces by 7.2% and 16.6%, and the in-plane shear strength reduces by 16.6% and 15.2% for the composites immersed into deionized water and artificial seawater, respectively. The main highlights of this paper are that it provides a more comprehensive mechanical properties test in combination with the microscopic characterization on a matrix and its composites to reveal the aging behavior of composites under a hygroscopic environment.

## 1. Introduction

Carbon fiber reinforced polymer (CFRP) has been widely used in many modern industrial fields in recent decades, such as in aerospace projects and wind turbine blades due to it high stiffness to weight ratio for weight-sensitive applications [1,2]. For marine engineering equipment, which is not relatively concerned with weight, the excellent durability of composites in marine environment is attractive [3]. In reality, glass fiber reinforced polymer (GFRP) whose modulus is lower than that of CFRP, has a long history in marine application (for example in boat hulls, military ships, submarines, etc.) [3,4]. When it is exposed to the hygrothermal environment for a long time, polymer and fiber reinforced polymer (FRP) will inevitably undergo a certain degree of aging, which results in the degradation of material properties [5,6,7,8,9,10]. Hygrothermal aging is a crucial factor that should be considered in overall structural design, especially for composites used in marine and naval engineering. The overestimation of aging influence hampers the cost effectiveness of the structure, while the underestimation of aging influence leads to challenges in terms of service safety. Thus, the effects of hygrothermal aging on structural performance should be appropriately quantified to design composites for wide applications while ensuring service safety.

To investigate the evolution of aging properties of polymer and FRP, the hygroscopic behavior of the materials should first be clarified. A large number of published papers have explored the hygroscopic behavior and mechanisms of polymer and FRP. Typically, it is believed that transportation of water molecules into material through diffusion-controlled and hydrolysis-controlled moisture absorption, and the specific moisture absorption mode, is determined by the molecular structure of the material and environmental factors [11,12]. Many studies have reported methods to accurately quantify the development of moisture uptakes of polymer; for example, one of the most common methods is using Fick’s law to establish the diffusion-controlled moisture absorption model as a function of time. However, many researchers have observed that polymer exposed to a hygrothermal environment exhibits non-Fickian diffusion model, such as two-stage moisture absorption curves [8,13,14]. Compared with pure resin, due to the embedment of fibers, FRP has more moisture absorption modes, such as capillary moisture absorption at the fiber–resin interface. At present, the emphases of most studies regarding the establishing the moisture absorption behavior of composites are still based on the Fickian diffusion model, and the corresponding diffusion coefficient is derived using the experimentally measured weight [15,16].

Researchers working on the design of engineering composite structures have been more focused on the degradation of material properties induced by liquid uptakes [9,17,18,19,20]. Water molecules as a source of hydrolysates and reaction potential for the hydrolytic group may trigger the breakage of molecular chains, formation of pores and micro-cracks, and then result in the decline of material strength [21]. The aging properties of GFRP or CFRP after moisture absorption have been broadly investigated. Grammatikos et al. [22] investigated the effect of hygrothermal aging on the durability of a coated ECR glass fiber reinforced polyester resin composite and found that increasing the aging temperature enhances the moisture absorption rate and the diffusion coefficient of water molecules. When the hygrothermal aging temperature was 80 °C, the specimen mass fell obviously due to resin decomposition and the leaching of the low molecular weight material into water. The tensile strength dominated by the fiber deteriorated insignificantly after hygrothermal aging, while the in-plane shear strength and modulus dominated by the resin matrix declined remarkably.

In structural engineering, epoxy resin and vinylester resin are extensively selected as the matrix of FRP [23]. Epoxy resin has excellent corrosion resistance and mechanical properties, and it is commonly used in marine engineering anti-corrosion coatings. Vinylester resin exhibiting low polarity and poor hydrophilic properties after curing and also has good durability in the hygrothermal environment [24]. Composites comprising different constituent material show a significant difference in the aging behavior. In marine and naval engineering structures, cost and performance are the main factors during the selection of structural materials. Considering the low cost, superior mechanical properties and excellent durability of glass fiber reinforced vinylester resin composites, it is preferred for application in marine and naval engineering structural equipment [25]. Some papers have reported the aging behavior of glass fiber reinforced unsaturated polyester resin composites [12,26,27]. These studies are concerned with the testing of tensile, bending, and interlaminar shear properties. The data and patterns reported in the literature show considerable variations, and the findings and conclusions for one material may not necessarily be applicable to other materials. With the wide application of GFRP in main load-bearing structures in marine and naval engineering, it is crucial to clarify its aging behavior in service. However, few studies have comprehensively focused on its durability in a marine environment.

The hygroscopic behavior of typical high strength glass fiber reinforced vinylester resin composites which were utilized in marine engineering equipment was investigated. Based on the service environment, the pure resin and GFRP specimens were manufactured and then immersed into two artificial seawater and deionized water tanks with a high temperature of 70 °C for 120 days for the gravimetric analysis and 90 days for the measurement of mechanical properties. The weight change and surface micro-morphologies of GFRP and pure resin specimens were checked, in combination with detecting the variations of resin molecular chain functional groups to identify the process of the mechanism of water penetration. Subsequently, the effects of hygrothermal aging on the mechanical properties of resin and composites were tested, including tensile strength, in-plane shear strength, and the compressive strength of GFRP. The typical marine environment test data of glass fiber reinforced vinylester resin composites are provided, which can provide insights for material selection reference data for durability design, corrosion mechanism research, and life prediction of composite structures, along with achieving service safety of marine and naval engineering composite equipment.

## 2. Materials and Methods

### 2.1. Materials

**Figure 1:**
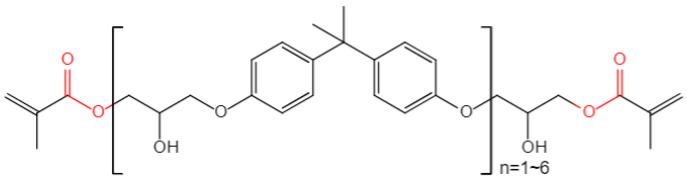
The structural formula of vinylester resin.

As stated in introduction, the types and combinations of constituent materials highly affect the aging behavior of composites. The investigated high strength glass fiber reinforced vinylester resin composites (referred to as GFRP below) were utilized in a practical marine engineering equipment for reasons of cost and performance. Conservative durability design was previously employed. To induce the weight of equipment and evaluate the real development of material properties during service, experimental investigations on the hygroscopic aging of CFRP are carried out here. The GFRP consists of 430LV vinylester resin from Nanjing Jinling DSM Co., Ltd. (Nanjing, China), and SW220C-100B high strength E-glass fiber fabric supplied by the Nanjing Fiberglass Research and Design Institute. The structural formula of vinylester resin is given in Figure 1. The curing agent is Aksu Butanox M50 which is composed of methyl ethyl ketone peroxide (MEKP), and the accelerator is cobalt iso-octoate. 

**Figure 2:**
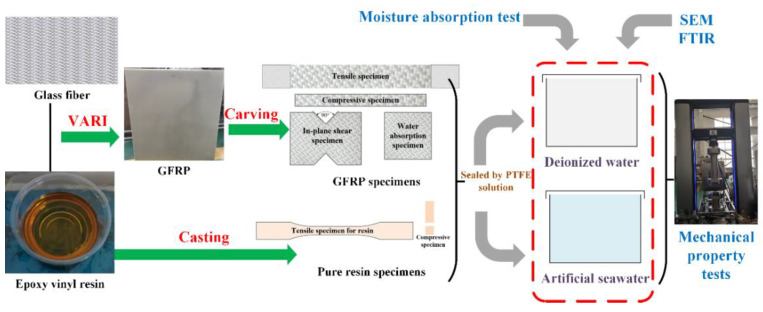
Flowchart of experimental investigation on aging behavior of resin and GFRP.

The deterioration of mechanical properties is a key indicator of aging, and the durability design of the composite structures is more concerned with the magnitude of degradation. Therefore, the mechanical properties of GFRP after aging were mainly tested below. Meanwhile, the test on mechanical properties of resin in combination with the observation of surface morphologies for specimens and identification of variations of functional groups for resin is also performed as the additional approaches to explain the aging behavior of GFRP. Figure 2 gives the next steps for investigating the hygroscopic behavior of resin and GFRP, including specimen fabrication and preparation, predefined hygroscopic conditions, and the mechanical properties measurement.

### 2.2. Specimen Preparation

**Figure 3:**
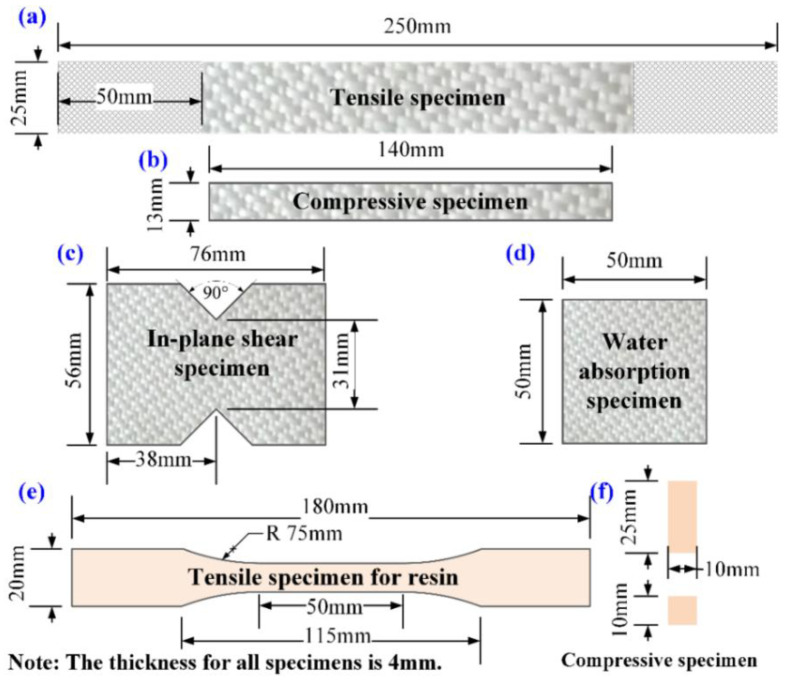
Standard specimens’ configuration: (**a**) tensile specimen for CFRP, (**b**) compressive specimen for CFRP, (**c**) in-plane shear specimen for CFRP, (**d**) water absorption specimen for CFRP, (**e**) tensile specimen for resin, (**f**) compressive specimen for resin.

Here, GFRP laminated plates were manufactured by vacuum-assisted resin infusion (VARI). After the resin was injected, the plates were cured at room temperature for 24 h, and post-cured at 100 °C for 4 h in a vacuum drying chamber. After demolding, the side edges of the GFRP laminated plates were cut and trimmed to obtain the predefined dimensions of 500 mm × 500 mm × 4 mm (length × width × thickness) for the next preparation of CFRP specimens. The fiber volume fraction of the finished GFRP laminated plates was 64%. The pure resin specimens were casted, and the curing condition was the same as for the GFRP laminates. The required specimens for different tests were cut from the CFRP laminated plates in accordance with the test standards using a numerical control carving machine from Roc machine electronic system engineering (Shanghai, China) Co., Ltd., as illustrated in Figure 3. The edge faces were polished by fine sandpaper and wiped by absolute ethyl alcohol. To remove the absorbed moisture during manufacturing, all specimens were placed in a vacuum with a temperature of 50 °C for 48 h. Subsequently, the edges of specimens were sealed by the room temperature curing polytetrafluoroethylene (PTFE) solution. After the PTFE was cured, all specimens were initially dried in a desiccator until their weight reached a constant value for the following use.

### 2.3. Hygroscopic Aging Environments

Two humid conditions were primarily encountered for the selected composite structures, i.e., the total immersion into seawater and contact with hot water from the boiler (the maximum possible temperature is 70 °C). Therefore, two experimental hygroscopic environments are set out as follows:

- Artificial seawater with a high temperature (abbreviated as AS)—the artificial seawater was produced based on ASTM-1141 by mixing NaCl, CaCl_2_, MgCl_2_, and NaHCO_3_, etc. The specimens were totally immersed into the medium with a high temperature of 70 °C;
- Deionized water with a high temperature (abbreviated as DW)—deionized water with a constant temperature 70 °C was prepared in the home-made climatic chamber to simulate the hygroscopic condition where composite components contacted with the hot water.

### 2.4. Characterization and Testing

#### 2.4.1. Moisture Absorption Behavior

The deterioration of mechanical properties is closely related to moisture absorption content, hence, the weight change in GFRP and resin were characterized through weighing the specimens and observing their surface morphology, as well as by detecting variations of functional groups of resin. 

Weight change measurements were carried out as follows: The moisture absorption behavior for GFRP and resin specimens were performed based on ASTM D5229/D5229M-2020 (moisture absorption properties and equilibrium conditioning of polymer matrix composite materials) and ASTM D570-2018 (standard test method for moisture absorption of plastics), respectively. The immersed specimens were taken out at the specified time intervals and the surficial water was wiped off using absorbent paper before measurement, followed by gravimetric analysis using electronic balance with a measurement precision of 0.1 mg. After weighing, the specimens were immediately placed back into the prescribed medium. The water uptake *M_t_* of a specimen was calculated by Equation (1) [28]. The time intervals for weighing the specimens are tabulated in Table 1.

(1) $$M_{i}=\left(\frac{w_{i}-w_{0}}{w_{0}}\right)\times 100\%$$

where $w_{i}$ and $w_{0}$ are the weight of the materials at aging time *t* and initial state, respectively. Fick’s law of diffusion generally enables the description of the process of water diffusion into materials and is used here to fit the measured water uptake data. Considering the thin thickness and protection of PTFE against water diffusion from the side edge, the expressions of one-dimensional diffusion are adopted as follows [28]:

(2) $$\frac{\partial C}{\partial t}=D\frac{d^{2}C}{dx^{2}}$$

where *C* is the concentration as a function of *t* and location *x*, and *D* is the diffusion coefficient. The solution to Equation (2) distinguishes the moisture absorption change to equilibrium state, and the commonly cited expression is as follows [28]:

(3) $$M_{t}=M_{\infty }\left\{1-\exp\left[-7.3{\left(\frac{Dt}{b^{2}}\right)}^{0.75}\right]\right\}$$

where *M_t_* and *M_∞_* are the water uptake by the material at *t* and equilibrium state, respectively. Here, *b* is equal to *h* (*h* is the thickness) for the material exposed on two sides to the same environment in this paper. The ratio of *M_t_* and *M_∞_* is proportional to square root of time in the initial stage for Fickian diffusion and, therefore, the diffusion coefficient can be calculated as follows [28]:

(4) $$D=\pi {\left(\frac{M_{t}h}{4M_{\infty }}\right)}^{2}\frac{1}{t}$$

During scanning electron microscopy (SEM), the surface micro-morphologies of specimens were observed using a JSM-IT300 scanning electron microscope.

During Fourier-transform infrared spectroscopy (FTIR), a Nicolet 6700 Fourier-transform infrared spectrometer was utilized to characterize the variation of resin molecular chain functional groups during immersion. Each specimen was scanned at a resolution of 2 cm^−^^1^ to obtain a spectrum in the wavenumber range 400~4000 cm^−1^.

#### 2.4.2. Mechanical Properties

The tensile strength, compressive strength, and in-plane strength of GFRP at the specified time intervals were studied, together with the comparative measurement of tensile and compressive properties for pure resin. Seven specimens were prepared per test condition, and the effective values after screening were recorded. For the testing of mechanical properties, 3–6 valid data are commonly retained after excluding the unacceptable failure modes and abnormal data. The time intervals for the measurement of mechanical properties are listed in Table 1. 

During the tensile properties test, the tensile properties of GRFP were investigated according to ASTM D3039/D3039M-2008 (the standard test method for tensile properties of polymer matrix composite materials) using the universal testing machine LE055(100KN) from Lishi (Shanghai, China) Instruments Co., Ltd. The tensile test of pure resin was in accordance with GB/T 2567-2021(the test methods for properties of resin casting body).

For the compressive properties test, the compressive properties of GFRP were investigated based on ASTM D6641/ D6641M-2014 (the test method for the compressive properties of polymer matrix composite materials using a combined loading compression, or CLC, test fixture). The compressive test of pure resin was in accordance with GB/T 2567-2021(the test methods for the properties of a resin casting body).

During the in-plane shear properties test, shear properties of GFRP were examined in compliance with ASTM D7078/D7078M-2012 (standard test method for shear properties of composite materials by v-notched rail shear method) using LE055(100KN) with a load speed of 2 mm/min. 

## 3. Results and Discussion

### 3.1. FTIR Results

**Figure 4:**
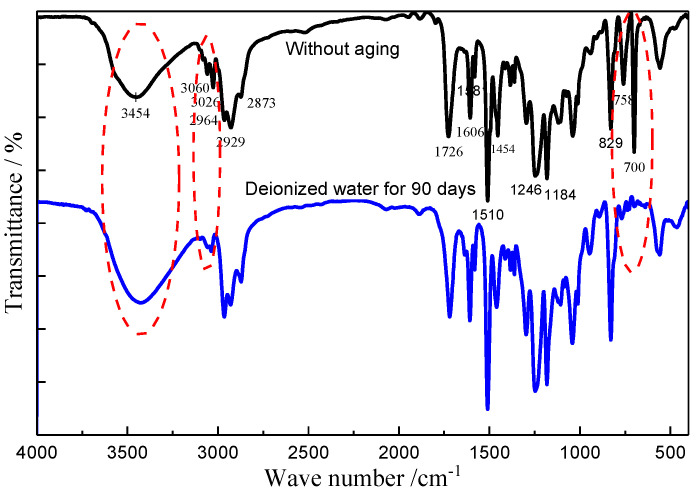
FTIR spectrogram for pure resin specimens.

**Figure 5:**
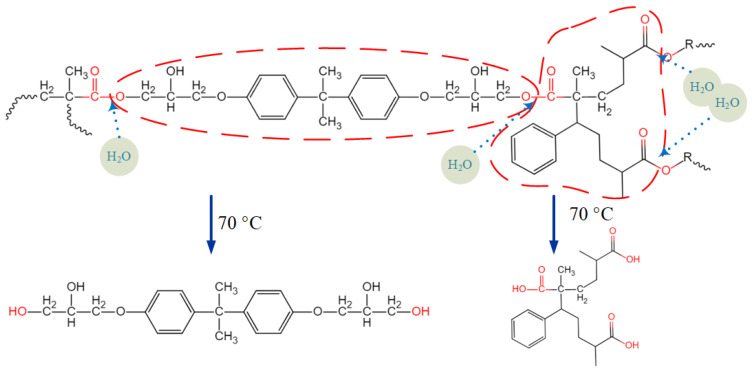
Schematic diagram of hydrolysis for vinylester resin.

After immersion for 90 days in DW, a large amount of white powder-like material appeared on the surface of the pure vinylester resin specimen. This surface powder, together with the scraped material from the surface of the unaged pure resin specimen, was analyzed by FTIR spectroscopy for comparison, and the results are shown in Figure 4. After aging for 90 days, FTIR absorption peaks of the white powdery substance were found to be highly overlapping with those of the unaged vinylester resin. Characteristic peaks were observed for the stretching vibration of the O-H group at 3454 cm^−1^, C-H stretching vibration of benzene ring at 3060 cm^−1^ and 3026 cm^−1^, C=O stretching vibration at 1726 cm^−1^, and stretching vibration absorption of benzene ring skeleton at 1606–1454 cm^−1^, and these were all consistent with the characteristic peaks for vinylester resin [10]. This indicated that the white powder on the surface after aging was not an impurity introduced during the experiment. After aging for 90 days, the O-H group stretching vibration characteristic peak at 3454 cm^−1^ was slightly enhanced. This was attributed to the hydrolysis of the ester bond in the resin molecular chain, which led to the generation of hydroxyl and carboxyl groups, thereby enhancing the O-H group stretching vibration characteristic peak [29]. The vinylester resin was subjected to free radical polymerization initiated by MEKP, which formed a cross-linked structure, and it had a small amount of styrene in the resin participating in the curing reaction [30], as shown in Figure 5. The methyl group at the α position of the ester bond of the resin molecular chain had a large steric hindrance effect and, therefore, vinylester resin had better hydrolysis resistance [24]. Due to the high temperature and long exposure time, the ester group was hydrolyzed into alcohol hydroxyl groups and carboxylic acids by water molecules; therefore, the monosubstituted benzene ring structure in the molecular chain, which was formed by the participation of styrene in the solidification, was lost in the medium by hydrolysis. The characteristic peaks of the C=C double bond did not appear in the IR spectra before and after aging. Considering that the C-H stretching vibration on the double bond in the range of 3075–3090 cm^−1^, the C=C stretching vibration in the range of 1620–1670 cm^−1^, and the C-H out-of-plane swing of vinyl in the range of 985–995 cm^−1^ did not appear in the spectrum, and the content of uncured vinyl-C=CH_2_ was considered extremely low. Hence, the out-of-plane bending vibration characteristic peaks of the monosubstituted benzene ring C-H at 758 and 700 cm^−1^ disappeared after 90 days of aging, and only the C-H out-of-plane bending vibration characteristic peak of the para-disubstituted benzene ring at 829 cm^−1^ was retained. Compared with the spectrogram before aging, the C-H stretching vibration characteristic peaks of benzene ring at 3060 and 3026 cm^−1^ were significantly weakened. The occurrence of hydrolysis and the loss of products caused the loss of mass of the specimens and, therefore, also affected the results of water absorption measured by the gravimetric method. 

### 3.2. Microscopic Morphology

**Figure 6:**
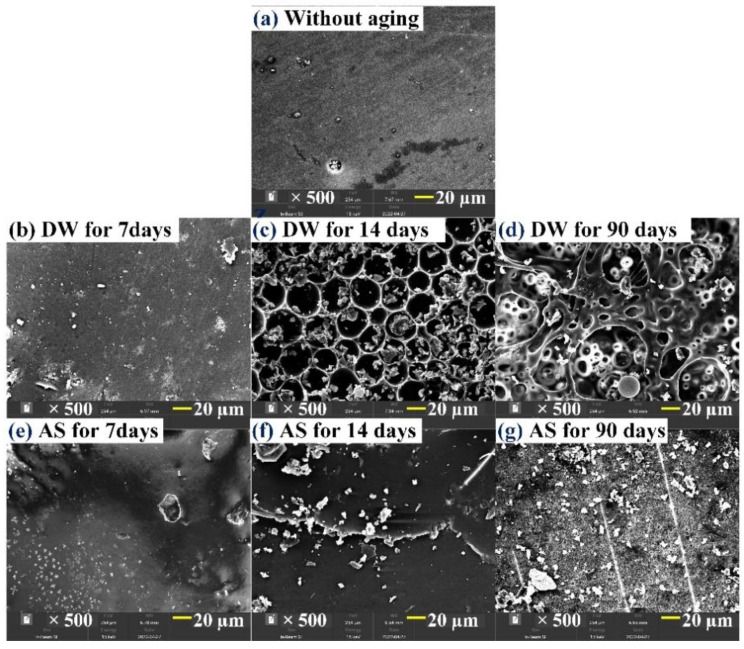
Evolution of surface morphology for pure resin immersed in DW and AS.

Figure 6 shows the surface morphology of pure resin specimens in DW and AS at different immersion periods. Before immersion, the surface of the resin was relatively flat, with a few small pits produced during preparation (Figure 6a). After immersion for 7 days in DW, the surface did not change significantly (Figure 6b). After immersion for 14 days, the surface was covered with regular circular pits (diameter = 20–30 μm), along with a large number of granular resin blocks (Figure 6c). Finally, after aging for 90 days, due to moisture diffusion, the pits gradually expanded and extended into the specimen to form porous and multi-channel structures (Figure 6d). Recalling the analyses in Figure 4, this was attributed to resin hydrolysis. Comparatively, after aging for 7 days in AS, the surface morphology of the specimen also varied insignificantly (Figure 6e). After aging for 14 days, micro-cracks were found in company with tiny granular resin particles adhered to surface of resin (Figure 6f). After aging for 90 days, the surface was covered with the tiny granular resin blocks and circular pits with a diameter of 1–2 μm (Figure 6g), signifying that hydrolysis also occurred for pure resin in AS. Compared with the AS environment, the resin lost more material in DW, suggesting that the resin was more prone to hydrolysis in DW. 

**Figure 7:**
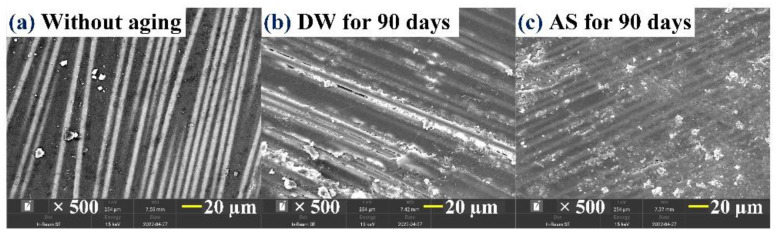
Evolution of surface morphology of GFRP immersed into DW and AS.

The surface morphology of GFRP before aging and after immersion for 90 days in DW and AS is depicted in Figure 7. Before immersion, the specimen surface was also flat. After aging for 90 days in DW, micro-cracks emerged in the glass fiber–resin interface and there was some material loss, demonstrating that hydrolysis took place in the resin matrix. However, the degree of hydrolysis for the resin matrix in DW was relatively weak compared with the pure resin in the same time period. After aging for 90 days in AS, a large number of tiny holes were seen on the resin surface near the local fiber–resin interface area, revealing that low-intensity hydrolysis also occurred to trigger material loss in this time. Comparing Figure 6 with Figure 7, it is easy to determine that GFRP did not form a porous and multi-channeled morphology in DW and AS and, therefore, the embedment of glass fiber inhibited the moisture diffusion in the resin matrix.

### 3.3. Weight Change

**Figure 8:**
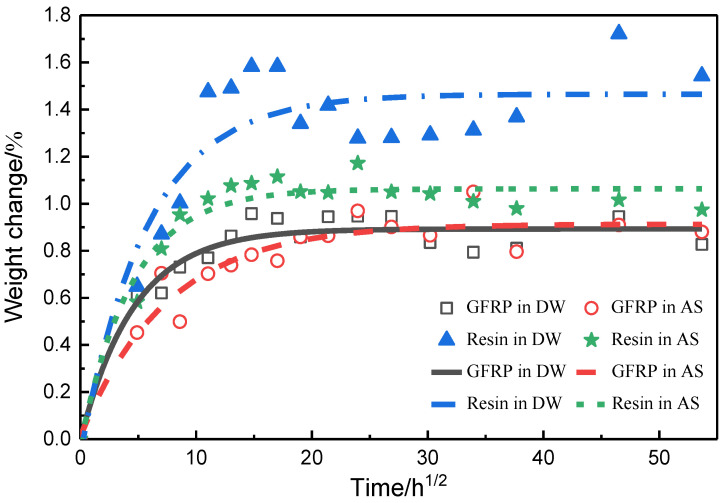
Weight change and fitted moisture absorption curves of pure resin and GFRP.

The moisture transportation into specimens can be considered as unidirectional diffusion given the thin thickness and protection of PTFE against diffusion from the side edge. Figure 8 displays the weight change in the pure resin and GFRP specimens, together with fitted curves using a nonlinear least square method based on the Fickian diffusion equation in Equation (3). The detailed parameters for fitted curves are tabulated in Table 2. The diffusion coefficients of pure resin specimens in DW and AS were 0.20 and 0.30 mm^2^/h, respectively, and the equilibrium water uptakes were 1.51% and 1.07%, respectively. By comparison, the diffusion coefficients of GFRP in DW and AS were 0.29 and 0.15 mm^2^/h, respectively, and the equilibrium water uptakes were 0.90% and 0.95%, respectively. The equilibrium water uptakes of GFRP were lower than those of the pure resin in the same hygrothermal environment because of the embedded glass fibers that curved the diffusion path of moisture in the resin and inhibited the diffusion of moisture in the resin matrix based on the above-mentioned analyses in Section 2.1 and Section 2.2. At the beginning of immersion, the weight of both pure resin and GFRP specimens increased rapidly due to diffusion-controlled moisture absorption. By combining Figure 4 with Figure 6, it could be obtained that the fluctuation in the mass of pure resin specimen after immersion for 12 days in DW was attributed to the higher hydrolysis-controlled moisture absorption and the corresponding material loss. Additionally, hydrolysis-controlled moisture absorption being the dominant factor firstly led to increase in mass, followed by the material loss causing the decrease in weight. Therefore, the weight change curve of pure resin in DW deviated remarkably from the Fickian behavior in the last region of curve. Meanwhile, the weight change in the last region of curve for pure resin in AS became slow after immersion for 12 days. Recalling the analysis in Figure 6, this was mainly because the resin underwent hydrolysis which resulted in the material loss. However, compared to the pure resin in DW, hydrolysis of pure resin in AS was not as strong. In DW and AS, the equilibrium mass of GFRP waved in the fitted Fickian curve due to the hydrophilicity of glass fiber, which facilitated the accumulation of a large amount of moisture at the fiber–resin interface. The hydrolysis of the resin matrix deteriorated the quality of the specimen, and also accelerated the capillary phenomenon due to the debonding of the interface. Therefore, the hygroscopic behavior of GFRP was influenced by multiple factors, including the free diffusion of water, the hydrolysis of the resin matrix, and the capillary phenomenon. 

### 3.4. Mechanical Properties 

**Figure 9:**
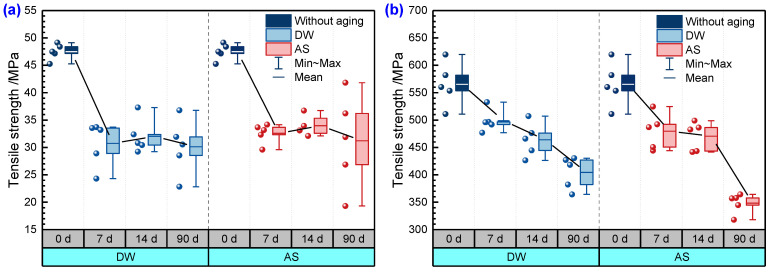
Tensile strength of (**a**) pure resin and (**b**) GFRP.

**Figure 10:**
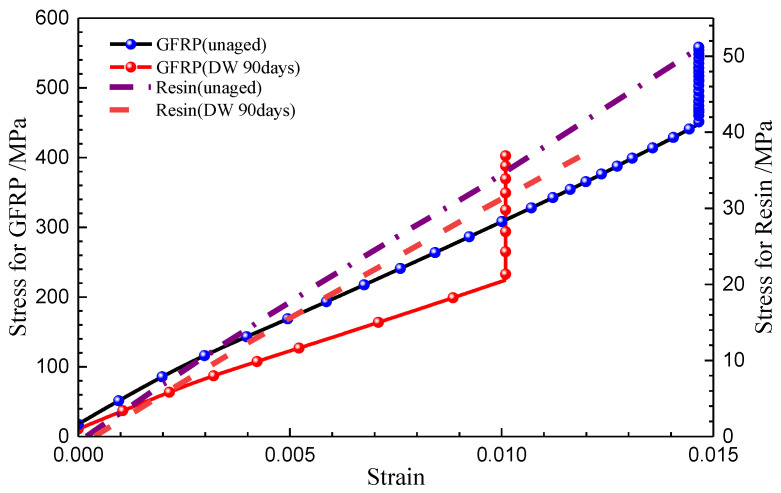
Typical stress–strain curves of GFRP under tensile load.

Figure 9 illustrates the variation of tensile strength with time for pure resin and GFRP specimens immersed in DW and AS. The tensile strength of the pure resin specimen decreased from 47 MPa to 31 MPa after immersion for 7 days in DW, with a loss of 35.3%. However, with the increase in immersion duration, the tensile strength varied inconsiderably in the subsequent immersion stage. In AS, the tensile strength of resin followed the same trend. It should be noted that after aging for 90 days in DW and AS, the scatter of the test data increased markedly. The tensile stress–strain curves of typical pure resin specimens before and after immersion are given in Figure 10, where the modulus is the slope of the curve. The modulus of resin fell after immersion. From the data given in Figure 8, the amount of moisture absorption was close to or higher than the fitted saturated moisture absorption after immersion for 7 days, and then the mass of the specimen kept relatively stable in the later period. The strength of resin depicted in Figure 9a also followed the same variation, and it could be assumed that the tensile strength of the pure resin was closely associated with the absorbed moisture. According to polymer physics, the plasticization caused by moisture absorption can lead to degradation of the material properties, and with longer immersion duration, occurrence of hydrolysis also induces the local defects in the polymer. The tensile strength reflects the performance of the weakest region of the material, and hydrolysis and moisture absorption both trigger the degradation of pure resin properties. The degree of deterioration varies asynchronously in different areas of the material and, therefore, the dispersion of the mechanical strength of the material increases, but the overall effect of hydrolysis on the tensile strength of the resin is limited after aging.

**Figure 11:**
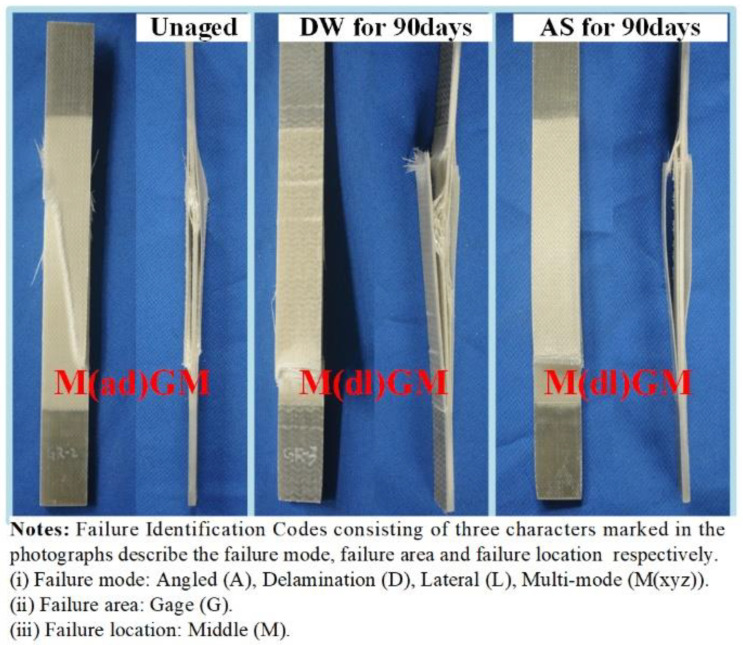
Typical failure modes of resin and GFRP specimens under the tensile test.

As a contrast, the tensile strength of GFRP also declined with the increase in immersion duration, and there was no significant difference between the effect of DW and AS environments on the tensile strength of GFRP after immersion for 14 days. After aging for 90 days, the tensile strength of GFRP in DW and AS decreased by 28.5% and 38.4%, respectively. The degradation in the strength of resin matrix caused a reduction in composite tensile strength; however, this was not the main reason for the reduction in the tensile properties of GFRP. According to the microscopic mechanics of the composites, the tensile modulus of GFRP in the fiber direction is dominated by the fiber modulus for the specific fiber volume fraction. The tensile strength of the fabric-reinforced composite depends mainly on the fiber strength and the properties of the fiber–resin interface. For composites in which the matrix elongation rate is less than the fiber elongation rate, the fiber strength is the dominant factor influencing the tensile strength of the composite. The typical tensile stress–strain curves for unaged GFPR and the GFPR aged for 90 days in DW are also shown in Figure 10. In accordance with the test standard, the tensile modulus of the composite was obtained from the slope between the two points, where the strain was 0.001 and 0.003, respectively. To prevent the vibration of the specimens from damaging the extensometer, the extensometer was removed before the specimen was damaged; therefore, the data of the strain before the damage was not collected, and the curve appeared as an upward vertical line. As shown in Figure 10, the tensile modulus of the aged composites was significantly lower than that before aging, indicating that the modulus of GFRP declined more significantly due to the immersion. The fracture elongation of resin was smaller than that of fiber and, therefore, the matrix cracked before the fiber during the damage to the composite, and the composite was completely destroyed when the strain reached the elongation of the fiber fracture. Figure 11 shows the failure modes of the typical GFRP tensile specimen, mainly in the form of superficial oblique fracture or lateral fracture (fiber fracture) with interlaminar delamination. Combined with the results in Figure 9b, Figure 10 and Figure 11, it can be inferred that the loss of GFRP tensile strength in DW and AS is mainly attributable to the degradation of the glass fiber strength.

**Figure 12:**
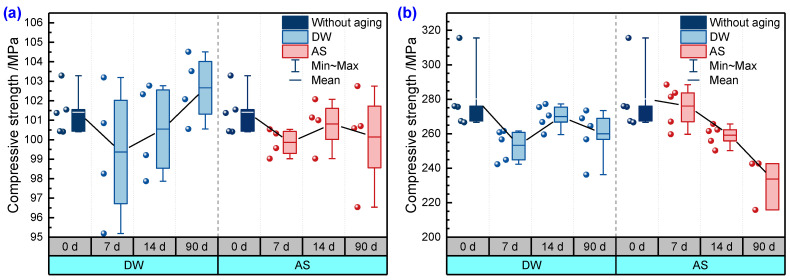
Compressive strength of (**a**) pure resin and (**b**) GFRP.

**Figure 13:**
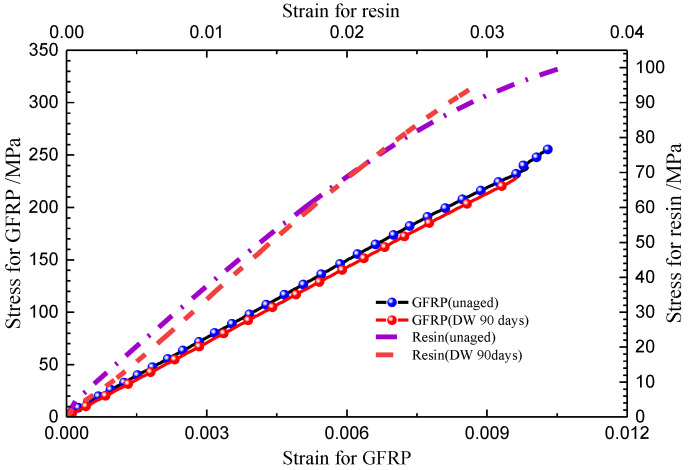
Typical stress–strain curves of resin and GFRP under a compressive load.

**Figure 14:**
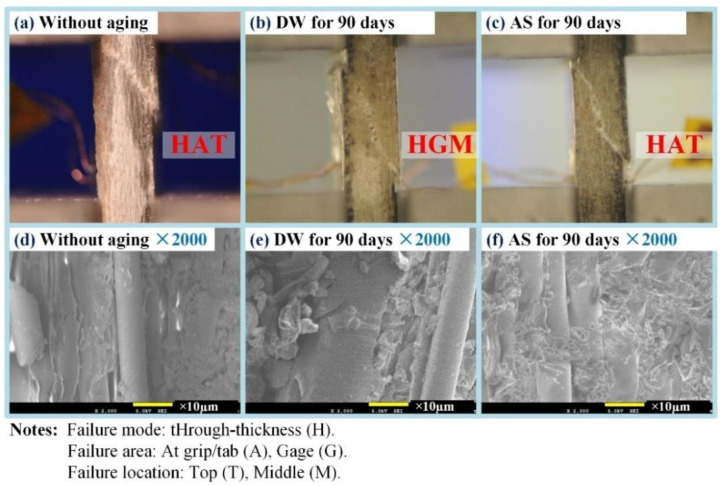
Typical failure modes of GFRP specimens under a compression test.

Figure 12 shows the compressive strength of pure resin and GFRP at different immersion durations in DW and AS. It is clear from Figure 12a that the compressive strength of pure resin was maintained at 100 MPa with an approximate fluctuation of 2 Mpa during immersion. The scatter of compressive strength increased with the increase in immersion duration. The compressive stress–strain curves of a typical pure resin before and after immersion in DW are shown in Figure 13. The compressive modulus of the resin calculated based on the slope fluctuated slightly after immersion for 90 days. From Figure 12b, it can be seen that the compressive strength of GFRP decreased slightly after aging. For GFRP, the compressive strength decreased by 7.2% and 16.6% after immersion for 90 days in DW and AS, respectively. Figure 13 shows the compressive stress–strain curves of GFRP before and after aging for 90 days in DW. According to the test standard, the compressive modulus was taken as the slope between two points (strains of 0.001 and 0.003). Similar to the stress–strain curve of resin specimens, the modulus of aged GFRP in DW only fell slightly compared to the unaged one. Figure 14 displays the failure modes and fracture morphologies of the compressive specimens of GFRP before and after immersion. The failure mode of the specimen before and after aging was mainly oblique fracture along the thickness direction. The fiber at the fracture cross-section before aging was smooth, and failure occurred near the clamping area; however, damage occurred mainly in the strain sheet and clamping area after aging. Meanwhile, it must be noted that resin particles were attached to the fiber surface after immersion for 90 days in AS, revealing the weakening of fiber–resin interface to some extent. Based on the microscopic mechanics of the composites, the compressive strength of GFRP was strongly influenced by the resin matrix, fiber–resin interface, interlaminar interface, and fiber properties. According to Figure 12a, the compressive strength of resin was not sensitive to the hygroscopic environment. Combining the stress–strain curve shown in Figure 13 and the fracture morphology shown in Figure 14, it can be inferred that the small fluctuation of GFRP (excluding the data of AS aging for 90 days) was attributable to the degradation of resin and fiber properties. After immersion for 90 days in AS, the failure was in the form of a weak interface of interface debonding, indicating the degradation of the resin–fiber interface property. After aging for 90 days in AS, only three valid data were obtained for compressive strength after excluding invalid data. Therefore, the effect of AS environment on compressive strength was further explored.

**Figure 15:**
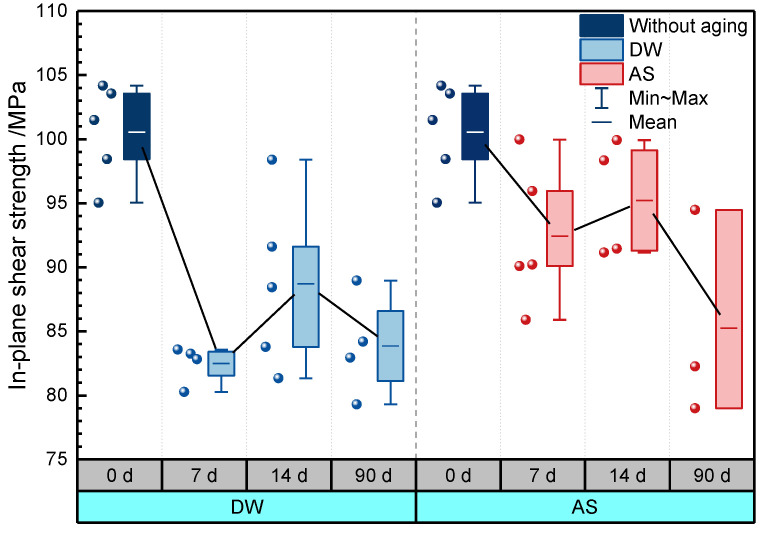
In-plane shear strength of GFRP.

**Figure 16:**
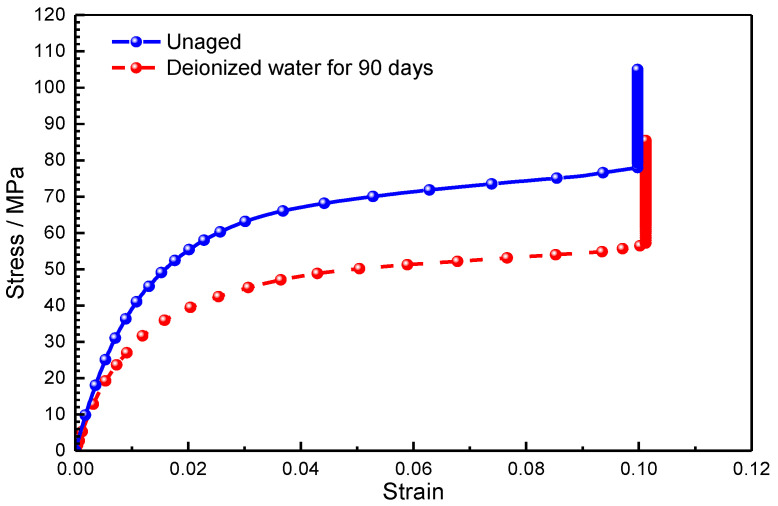
Typical stress–strain curves of GFRP under in-plane shear load.

**Figure 17:**
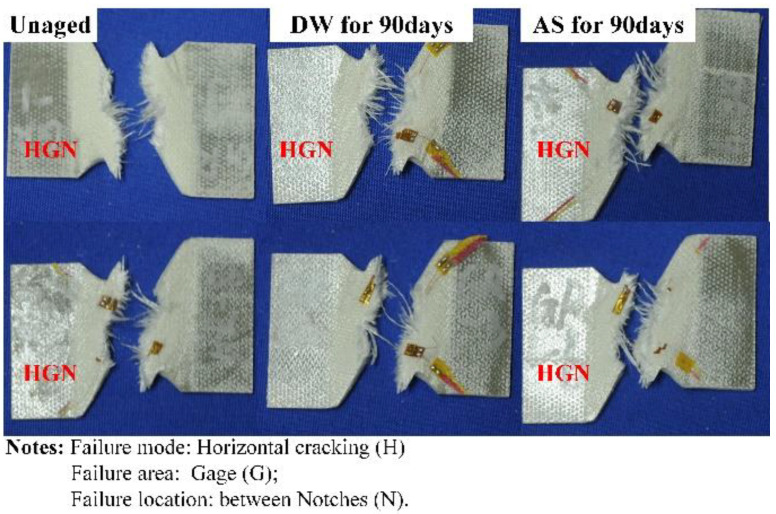
Typical failure modes of GFRP specimens under in-plane shear test.

Figure 15 displays the variation of in-plane shear strength of GFRP with immersion duration in DW and AS. The in-plane shear strength decreased from 101 MPa to 82 MPa after immersion for 7 days (decrease of 18.0%) in DW, and it fluctuated around 85 MPa in the subsequent immersion stage. In AS, the in-plane shear strength degraded with the increasing immersion time. After immersion for 90 days, the in-plane shear strength reduced by 15.2%, and there were only slight differences in the magnitude of reduction compared to the data from the specimens immersed into DW for 90 days. Figure 16 gives the typical GFRP stress–strain curve under in-plane shear loading before and after immersion. The in-plane shear modulus was obtained from the slope between two points (strains of 0.002 and 0.006, respectively) and it can be seen that the same reduction in in-plane shear modulus after immersion for 90 days in DW and AS. Figure 17 shows the typical failure mode of GFRP specimens in in-plane shear test. Before and after immersion, transverse cracks formed in the area between the two “V”-shaped notches. Consistent with microscopic composite mechanics, the in-plane shear strength depends on the properties of the fiber, resin, and the fiber–resin interface. Based on the analyses from Figure 9 and Figure 10, the decrease in the in-plane shear strength at the early stage of immersion was attributed to the deterioration of resin and fiber strength. Meanwhile, a smaller fluctuation of in-plane shear strength in DW in the later stage of immersion period was in line with the slight change in in-plane shear strength in DW at the same period, and additionally demonstrated that the properties of the fiber–resin interface degraded inconsiderably in the late stage of DW immersion. As with the deterioration of compressive strength, the decline in the in-plane shear strength after 90 days of AS aging was caused by the deterioration of the properties of the fiber–resin interface.

**Figure 18:**
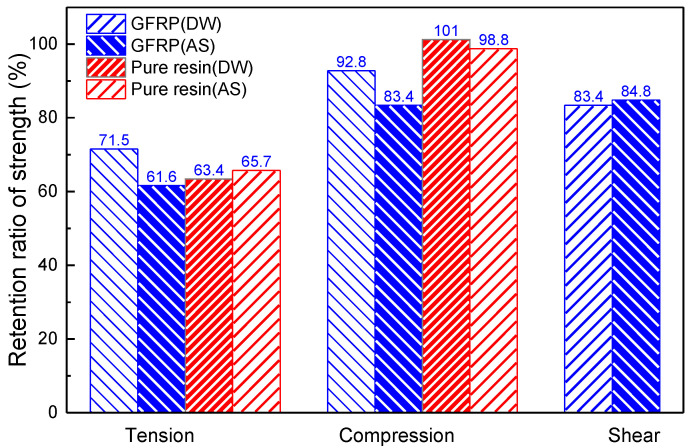
Retention ratio of strength for GFRP and pure resin specimens after aging for 90 days.

Figure 18 shows the strength retention ratio of pure resin and GFRP after immersion for 90 days in DW and AS. The selected hygroscopic environments had significant effects on the tensile strength of the specimens. Specifically, the tensile strength of the pure resin and GFRP specimens decreased by up to 36.6% and 38.4%, respectively, after immersion in DW and AS baths for 90 days. The in-plane shear strength of GFRP decreased by up to 16.6% after immersion for 90 days. The compressive strength of pure resin was not influenced by the hygrothermal environment, and the compressive strength of GFRP decreased by up to 16.6% after immersion for 90 days.

## 4. Conclusions

The weight change in vinylester resin and its high strength glass fiber reinforced vinylester resin composites immersed in DW and AS were examined, together with the observation of surface morphologies and changes in the infrared spectrum. Meanwhile, the comprehensive mechanical properties of resin and GFRP after hygroscopic aging were tested.

The results from the FTIR analysis, microscopic morphology observation, and weight change measurement show that vinylester resin undergoes severe hydrolysis during immersion in DW and that molecular chains breakage occurs in the pure resin specimen due to hydrolysis. Comparatively, the embedment of glass fiber inhibits the hydrolysis of rgw resin matrix in composites, resulting in lower equilibrium water uptakes of GFRP than those of the pure resin in the same hygrothermal environment.

The test on the mechanical properties of pure vinylester resin specimens reveals that the tensile strength of pure resin respectively decreases by 36.6% and 34.3% after immersion for 90 days in DW and AS, while the compressive strength of pure resin consistently dwells at 100 ± 2 MPa. As a contrast, the tensile strength of GFRP decreases by 28.5% and 38.4%, the compressive strength of GFRP reduces by 7.2% and 16.6%, and the in-plane shear strength of GFRP reduces by 16.6% and 15.2% after immersion for 90 days in DW and AS, respectively. From the experimental data, it can be concluded that tensile strength of resin and composites is strongly affected by the environments of DW and AS. The provided comprehensive test data of glass fiber reinforced vinylester resin composites in a typical marine environment contribute to the material selection for durability design, corrosion mechanism research, and the life prediction of composite structures.

## Figures and Tables

**Table 1:** Aging environments and prescribed time for experimental measurement.

Terms	Hygroscopic Environment	
	Deionized Water with 70 °C (DW)	Artificial Seawater with 70 °C (AS)
Medium	Deionized water	Artificial seawater (NaCl, MgCl_2_, etc.)
Temperature	70 °C	70 °C
Time for weighing specimens (days)	1, 2, 3, 5, 7, 9, 12, 15, 19, 23, 30, 38, 48, 60, 90, 120.	1, 2, 3, 5, 7, 9, 12, 15, 19, 23, 30, 38, 48, 60, 90, 120.
Time for mechanical testing (days)	7, 14, 90.	7, 14, 90.

**Table 2:** The non-linear fitted parameters for Fickian diffusion.

Items	Effective Equilibrium Water Uptake $\left(M_{\infty }\right)$/%	Diffusion Coefficient (*D*)/mm^2^·h^−1^	Coefficient of Determination (*R^2^*)
Resin in DW	1.50624	0.19909	0.81803
Resin in AS	1.07048	0.30306	0.91841
GFRP in DW	0.90124	0.28912	0.91565
GFRP in AS	0.94617	0.15499	0.90629

## Data Availability

Not applicable.
